# Two-Year Radiological Fusion Outcomes Following Biportal Endoscopic Transforaminal Lumbar Interbody Fusion Using Banana-Shaped Interbody Cages

**DOI:** 10.3390/jcm14228091

**Published:** 2025-11-14

**Authors:** Sang-Bum Kim, Dong-Hwan Kim, Daehee Choi, Ja-Yeong Yoon

**Affiliations:** Department of Orthopedic Surgery, Chungnam National University Sejong Hospital, Sejong 30099, Republic of Korea; sangbumos@me.com (S.-B.K.); kidhw411@gmail.com (D.-H.K.);

**Keywords:** endoscopic spinal surgery, transforaminal lumbar interbody fusion, radiological fusion, porous titanium cage, interbody fusion, minimally invasive spine surgery, I-factor

## Abstract

**Background**: Biportal endoscopic transforaminal lumbar interbody fusion (BESS-TLIF) is an emerging minimally invasive technique. This study aimed to evaluate the two-year radiological fusion outcomes of single-level BESS-TLIF using a specific banana-shaped, porous titanium interbody cage. **Methods**: This retrospective study reviewed 51 patients who underwent the specified procedure. The primary endpoint was the radiological fusion rate, assessed by computed tomography (CT) over 24 months using a three-grade system. Factors influencing fusion, particularly bone graft composition (demineralized bone matrix [DBM] only vs. DBM with I-factor), were also analyzed. **Results**: The final complete fusion rate at two years was 96.1% (49/51; 95% Confidence Interval (CI), 86.5–99.5%). Bony fusion occurred predominantly in the posterior and intracage regions. The only significant factor influencing fusion was the bone graft material. The ‘DBM with I-factor’ group achieved complete fusion significantly faster than the ‘DBM only’ group (log-rank test, *p* < 0.001), with a higher final fusion rate (100% vs. 83.3%, *p* = 0.045). **Conclusions**: Single-level BESS-TLIF using a banana-shaped, porous titanium cage provides favourable two-year radiological fusion rates. The selective addition of I-factor as an osteoinductive supplement can significantly accelerate the time to achieve solid arthrodesis.

## 1. Introduction

Lumbar degenerative disease is one of the most common causes of low back pain and radiculopathy. For patients who fail to improve despite an adequate course of conservative treatment, spinal fusion has become an effective surgical option [1]. While traditional open transforaminal or posterior lumbar interbody fusion (TLIF/PLIF) has shown stable results, it is associated with significant paraspinal muscle injury, which can lead to increased postoperative pain and a prolonged recovery period. To overcome these drawbacks, minimally invasive spine surgery (MISS) techniques were developed, and more recently, biportal endoscopic spinal surgery (BESS) has emerged as an even more advanced minimally invasive approach. BESS-TLIF has been reported to have several clinical advantages, including minimal muscle damage and faster postoperative recovery [2]. However, the evidence regarding radiological fusion, one of the most critical criteria for the long-term success of BESS-TLIF, remains insufficient. Many existing studies have reported on relatively short-term follow-up periods of around 12 months or have relied on plain radiographs, which have limited accuracy for assessing bony fusion [3]. Furthermore, previous studies have often included heterogeneous patient cohorts with various types of interbody cages or multi-level fusions, making it difficult to evaluate the pure outcomes of a specific surgical procedure and implant combination [3,4].

In particular, recently developed 3D-printed porous titanium cages have been suggested to promote higher fusion rates by enhancing osseointegration compared to traditional materials like polyetheretherketone (PEEK) [5]. However, there is a lack of data on the long-term fusion patterns when these modern cages are used in BESS-TLIF procedures.

Therefore, the purpose of this study was to conduct a detailed analysis of the long-term, 24-month radiological fusion outcomes using computed tomography (CT), the gold standard for fusion assessment, in patients who underwent single-level BESS-TLIF with a single type of porous titanium cage (EIT Cellular Titanium TLIF Cage). We also aimed to evaluate the factors that may influence the fusion status at 24 months.

## 2. Materials and Methods

### 2.1. Patient Population

This retrospective study was approved by the Institutional Review Board of our institution (IRB No. 2025-07-005) and was conducted in accordance with the ethical principles of the Declaration of Helsinki. The requirement for written informed consent was waived due to the study’s retrospective nature.

**Figure 1:**
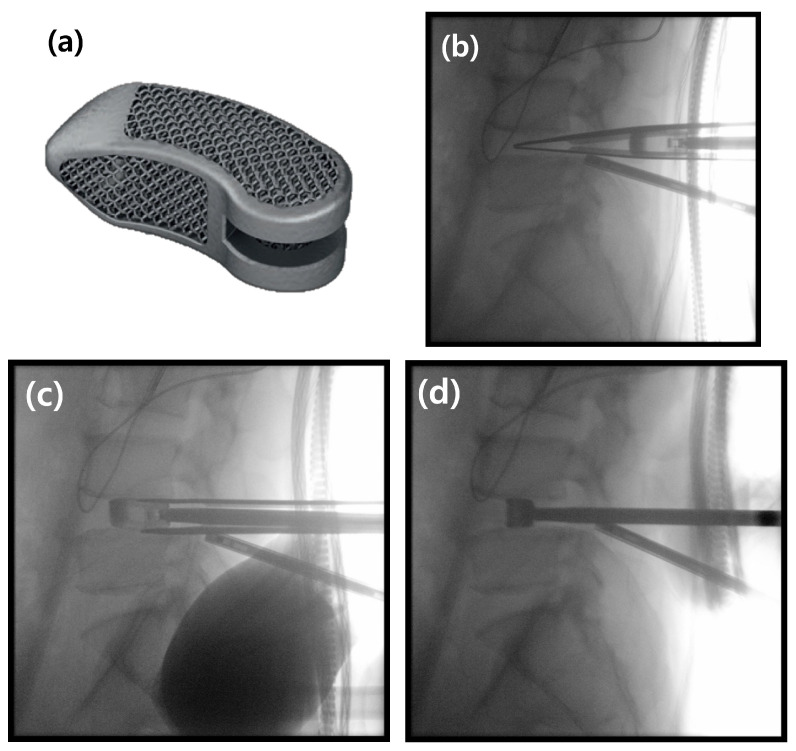
The banana-shaped EIT Cellular Titanium TLIF Cage and the intraoperative “insert-and-rotate” insertion technique. (**a**) Photograph of the EIT Cellular Titanium TLIF Cage, showing its porous, trabecular structure. (**b**) Intraoperative lateral C-arm image demonstrating the initial insertion of the cage into the posterior aspect of the disc space. (**c**) The “insert-and-rotate” maneuver is performed on the lateral view; the inserter handle is pivoted medially, which initiates the rotation of the cage. (**d**) Final lateral C-arm image confirming the cage has been rotated 90 degrees into its correct transverse position across the interbody space.

This study was designed to retrospectively evaluate patients who underwent primary single-level BESS-TLIF using the EIT Cellular Titanium TLIF Cage (DePuy Synthes, Johnson & Johnson; Tuttlingen, Germany) (Figure 1). We reviewed the medical records of patients who received this specific implant for degenerative lumbar disease. The inclusion criteria were: (1) a diagnosis of lumbar foraminal stenosis or degenerative spondylolisthesis of Meyerding grade I or less (representing low-grade instability); (2) persistent symptoms despite at least three months of conservative treatment; and (3) the availability of complete clinical and radiological data for a minimum of two years postoperatively.

Patients were excluded from the study if they had a history of previous surgery at the index level, required multi-level fusion, or underwent a hybrid surgical procedure combining different techniques. Patients who presented with other pathological conditions such as active infection, spinal tumours, metastatic disease, or acute fractures were also excluded. A total of 55 patients initially met these criteria. During the follow-up period, four patients (7.3%) developed significant cage subsidence (>3 mm), which was considered a procedural complication. As the resultant narrowing of the disc space also confounds the radiological assessment of bony fusion, these 4 patients were excluded from the primary per-protocol analysis of fusion grading. Therefore, a final cohort of 51 patients, comprising 22 males and 29 females, was included in the detailed radiological fusion analysis. All patients underwent follow-up CT scans at 3, 6, 12, and 24 months after surgery.

### 2.2. Surgical Procedures

All surgical procedures were performed under general anesthesia. Patients were positioned prone on a Jackson table, and the surgical field was prepared and draped in a standard sterile fashion for endoscopic spine surgery. The surgical approach was performed on the side with predominant radicular pain. In cases of bilateral symptoms, the left-sided approach was typically chosen to facilitate instrument handling for the right-handed surgeon.

Under C-arm fluoroscopic guidance, the operative disc level was identified. The pedicles of the superior and inferior vertebrae of the operative segment on the side of the surgical approach were then identified and marked on the skin. Two horizontal skin incisions, approximately 1.5 to 2.0 cm in length, were made directly over these pedicle markings. These incisions served as the entry portals for both the endoscopic procedure and the subsequent ipsilateral pedicle screw fixation.

Following the incisions, a sequential dilator was introduced to bluntly dissect the multifidus muscle, creating working channels down to the lamina. Initially, the upper incision served as the viewing portal and the lower as the working portal. An arthroscope was introduced through the upper portal, and a bipolar radiofrequency probe was inserted through the lower portal. This established the initial triangulation and allowed for soft tissue dissection and hemostasis. A clear surgical field was maintained throughout the procedure by ensuring a smooth and continuous outflow of saline irrigation.

**Figure 2:**
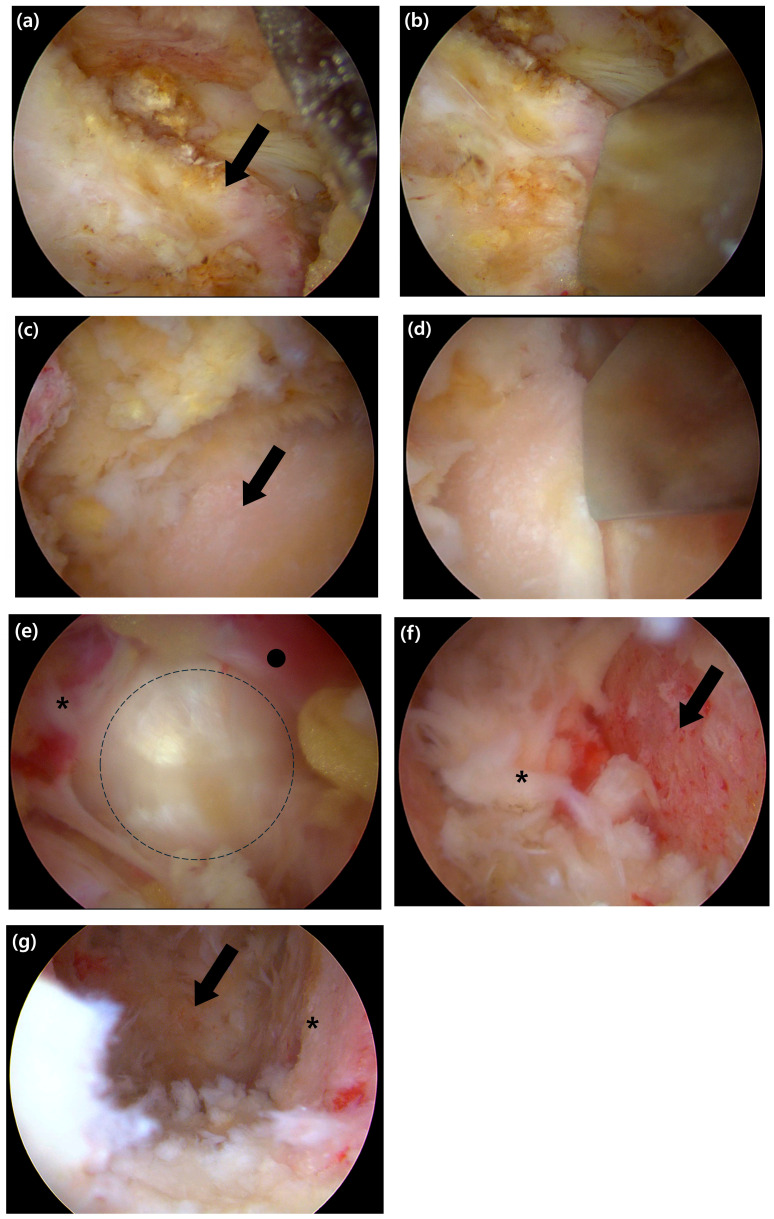
Intraoperative endoscopic views of the key steps for decompression and endplate preparation. (**a**) Endoscopic view showing the exposed inferior articular process (IAP) of the superior lamina (black arrow). (**b**) The IAP is resected using an osteotome. (**c**) After resection of the IAP, the superior articular process (SAP) of the inferior lamina is visualized (black arrow). (**d**) Resection of the SAP is performed with an osteotome to achieve foraminal decompression. (**e**) View after complete removal of the ligamentum flavum, showing the exposed annulus fibrosus. The exiting nerve root (asterisk), traversing nerve root (black circle), and the disc space (the dashed outline) are indicated. (**f**) The superior bony endplate of the inferior vertebra (black arrow) is revealed after meticulous stripping of the cartilaginous layer; a remnant disc fragment is marked with an asterisk. (**g**) Final view after endplate preparation, showing the prepared bony margin (asterisk) and the visualized anterior margin of the annulus (arrow), which confirms the anterior boundary of the disc space.

Once a clear surgical field was established, an ipsilateral partial laminotomy was first performed and then extended via an undercutting technique to achieve bilateral decompression. Following this decompression, the ipsilateral inferior articular process (IAP) of the superior lamina was resected using an osteotome for autologous bone grafting (Figure 2a,b). Then, the superior articular process (SAP) of the inferior lamina was partially resected to fully expose the foraminal area (Figure 2c,d). This primary bony resection with the osteotome was performed while preserving the ligamentum flavum, which acted as a natural barrier to protect the dura and nerve roots. A high-speed burr was then used to remove any remaining sharp edges and meticulously shape a smooth and safe transforaminal pathway for the subsequent steps.

Following the completion of the bony work, the ligamentum flavum was completely removed to achieve full decompression. Using a curette and a rotating Kerrison punch, the ligament was first gently detached from the dura and excised on the ipsilateral side. The decompression was then extended to the contralateral side via a sublaminar approach. The ligament was removed either en bloc or in large fragments, ensuring the thorough decompression of the thecal sac and the traversing nerve root (Figure 2e).

Once neural decompression was completed, an annulotomy was performed using an annular knife (Figure 2f). A thorough discectomy was then carried out to create sufficient space for the interbody cage and bone graft. The nucleus pulposus was removed using a combination of curettes, pituitary forceps, and smooth-edged interbody disc reamers. Under magnified endoscopic vision, the cartilaginous endplates were then meticulously stripped from the underlying bone, which allowed for verification of complete residual disc removal and confirmation of endplate integrity (Figure 2g). This entire removal process was continued until the transverse fibres of the anterior longitudinal ligament (ALL) were clearly visualized, signifying the anterior boundary of the disc space.

**Figure 3:**
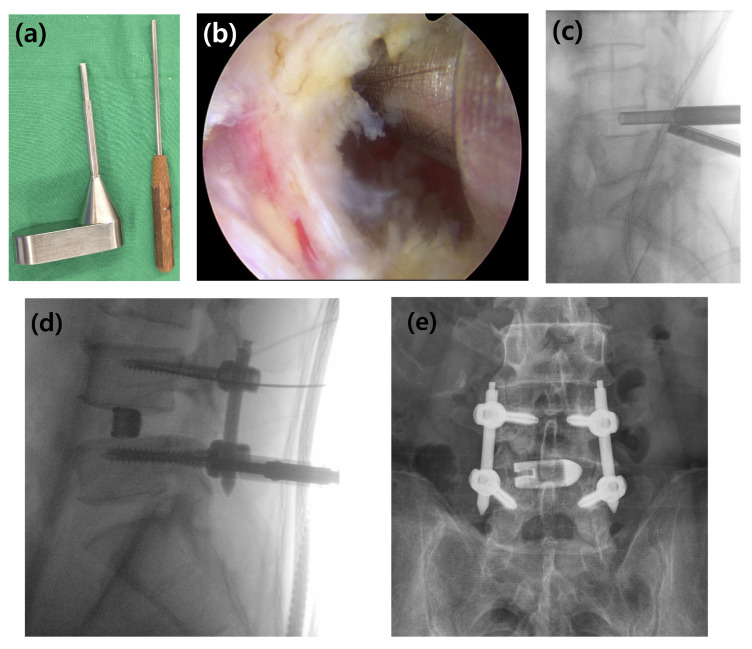
Surgical instruments and the procedure for interbody bone graft delivery. (**a**) The specialized bone graft funnel and the corresponding impactor used to deliver the graft material into the disc space. (**b**) Intraoperative endoscopic view showing the funnel correctly positioned at the entrance of the prepared disc space. (**c**) Lateral C-arm image confirming the position of the funnel and demonstrating the delivery of the bone graft material into the interbody space. (**d**) Intraoperative lateral C-arm image showing the insertion of percutaneous pedicle screws for posterior stabilization after cage placement. (**e**) Final anteroposterior radiograph showing the completed construct with the cage and pedicle screws.

Following endplate preparation, serial trials and interbody shapers were used to determine the optimal cage size that would ensure a press-fit and restore disc height. The saline irrigation was then temporarily paused to prevent graft washout. For interbody grafting, a composite of local autograft harvested during decompression and DBX Putty (demineralized bone matrix [DBM]; MTF Biologics; Edison, NJ, USA) was utilized. The local autograft provided essential osteogenic cells, while DBM served as an osteoconductive graft extender to supplement the often-limited autograft volume in MISS [6]. To further enhance the biological stimulus for fusion, an osteoinductive agent was considered. While bone morphogenetic protein-2 (BMP-2) is a potent option, its use within the interbody space has been associated with significant complications, such as ectopic bone formation, vertebral osteolysis, and inflammatory responses [5]. Therefore, I-factor bone graft (Cerapedix; Westminster, CO, USA), a synthetic peptide (P-15) with a favourable safety profile for interbody applications, was used as an osteoinductive supplement in this study [5,7,8]. Due to the additional cost, I-factor was administered only to those patients who provided specific informed consent. This material was delivered and compacted into the anterior portion of the disc space using a specialized funnel (Figure 3a–c).

The cage of the selected size was then packed with the remaining bone graft mixture. Throughout the insertion, the exiting and traversing nerve roots were protected under direct endoscopic visualization. The cage was inserted using an “insert-and-rotate” technique; it was first introduced into the posterior disc space, after which the inserter handle was pivoted medially. This maneuver allowed the banana-shaped cage to rotate into a transverse orientation as it was advanced across the disc space [9]. The cage was then gently impacted into its final position, and its placement was confirmed with C-arm fluoroscopy.

Posterior stabilization was then achieved with percutaneous pedicle screw fixation. Under fluoroscopic guidance, cannulated pedicle screws were inserted bilaterally; the contralateral screws were placed through separate incisions, while the ipsilateral screws were inserted through the established endoscopic portals. Rods were passed percutaneously, contoured to the desired lordosis, and the entire construct was secured (Figure 3d,e). After meticulous hemostasis was achieved with the radiofrequency probe, a drainage tube was placed at the surgical site. The fascia and skin incisions were then closed in a layered fashion.

### 2.3. Postoperative Management

Postoperatively, all patients were required to wear a thoracolumbar sacral orthosis (TLSO) for a period of six months to provide external stability. To further promote bony fusion, a 6-month course of teriparatide (Terrosa^®^; Gedeon Richter Plc.; Budapest, Hungary; 20 μg daily, subcutaneous injection) was administered. This 6-month duration was strategically chosen to maximize the therapeutic effect within teriparatide’s peak “anabolic window,” which is the initial phase when its bone-forming activity significantly exceeds bone resorption, while also maintaining cost-effectiveness for the patient [10,11].

### 2.4. Clinical and Radiological Assessment

#### 2.4.1. Primary Endpoint: Radiological Fusion Assessment

**Figure 4:**
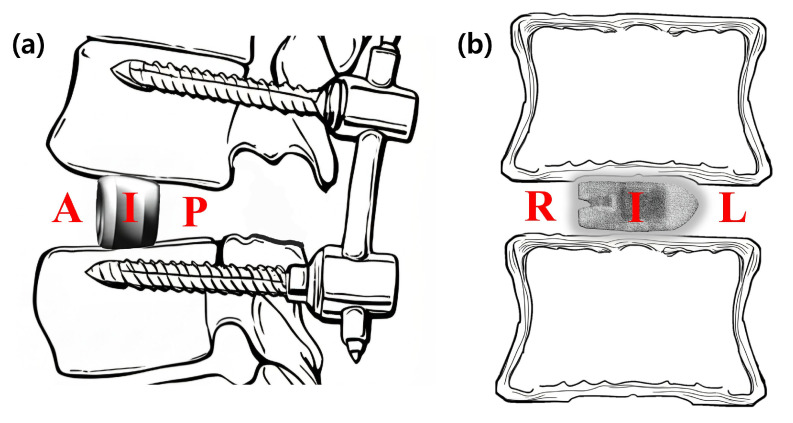
Schematic diagrams illustrating the classification of fusion location. The location of new bone formation was assessed relative to the interbody cage on multi-planar CT images. (**a**) On the sagittal plane, fusion was evaluated in three zones: anterior to the cage (A), inside the cage (I), and posterior to the cage (P). (**b**) On the coronal plane, fusion was evaluated in three zones: right of the cage (R), inside the cage (I), and left of the cage (L). The laterality (left/right) was determined based on the radiological orientation of the CT image.

The primary endpoint of this study was the radiological fusion rate, assessed using multi-planar reconstructed CT images obtained at 3, 6, 12, and 24 months postoperatively. To provide a detailed analysis, the location of new bone formation was classified based on its position relative to the cage. On sagittal plane images, the locations were defined as (A) anterior to the cage, (I) inside the cage, and (P) posterior to the cage. On coronal plane images, they were defined as (L) left of the cage, (I) inside the cage, and (R) right of the cage (Figure 4).

**Figure 5:**
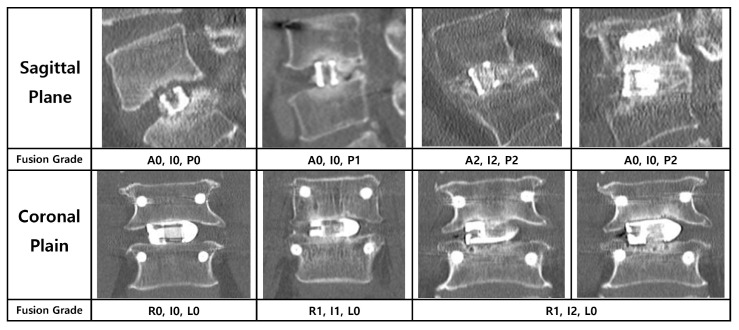
Radiological criteria and representative CT images for the three-grade fusion grading system. The figure illustrates the grading system used to assess bony fusion on multi-planar CT images, with representative examples provided for both the sagittal (left column) and coronal (right column) planes. Fusion was categorized into three grades: Grade 0 (Non-union) was defined as the complete absence of new bone formation; Grade 1 (Partial Fusion) as the presence of bone growth without a continuous bridging trabecular bone between the vertebral bodies; and Grade 2 (Complete Fusion) as the formation of a continuous, solid bone bridge. For the purpose of the final analysis in this study, only Grade 2 was considered a successful fusion.

The degree of fusion was evaluated using a simplified three-grade grading system. While the classification described by Bridwell et al. is a foundational standard, we adopted a more distinct definition for clarity, inspired by the methodology of a prior study on PLIF fusion rates [12]. Fusion was graded as follows: Grade 0 (Non-union) was defined as the complete absence of bone growth; Grade 1 (Partial Fusion) as the presence of bone growth without a continuous bone bridge connecting the superior and inferior vertebral bodies; and Grade 2 (Complete Fusion) as the formation of a continuous, solid bone bridge. For the final analysis, Grade 2 was considered a successful fusion (Figure 5).

#### 2.4.2. Secondary Endpoints: Analysis of Factors Influencing Fusion

As a secondary analysis, we evaluated the influence of several variables on the radiological fusion outcome at 24 months. These variables included patient-related factors such as age and bone mineral density (BMD), and surgery-related factors such as implanted cage height, lordotic angle, and final cage position. The composition of the fusion materials, as previously described, was also analyzed as a factor, categorized into two groups: local autograft with DBM only, or with the addition of I-factor.

### 2.5. Statistical Analysis

All statistical analyses were performed using Python (version 3.9; Python Software Foundation; Wilmington, DE, USA) with the Pandas (version 1.5.3), SciPy (version 1.10.1), and Lifelines (version 0.27.8) libraries. Baseline characteristics were summarized using descriptive statistics (mean ± standard deviation (SD) or *n* [%]). The primary endpoint was the radiological fusion status (Grade 0, 1, or 2), assessed over 24 months. To identify factors influencing fusion, Fisher’s exact test and the Kruskal–Wallis H test were used for univariable comparisons. Time-to-fusion events were analyzed using the Kaplan–Meier method with the log-rank test. The frequency of fusion at different anatomical locations was compared using Cochran’s Q test with post hoc McNemar tests. A *p*-value < 0.05 was considered statistically significant.

## 3. Results

### 3.1. Baseline Demographic and Surgical Characteristics

The mean age of the cohort was 70.0 ± 9.7 years, with a mean BMD T-score of −1.7 ± 1.1 (Table 1). The study population consisted of 22 males (43.1%) and 29 females (56.9%). The most common surgical level was L4–5 (*n* = 27, 52.9%), followed by L5–S1 (*n* = 13, 25.5%). A bone graft mixture including I-factor was used in the majority of patients (*n* = 39, 76.5%). For the interbody cage, the most frequently used height was 12 mm (*n* = 20, 39.2%), and the most common lordotic angle was 8° (*n* = 38, 74.5%).

### 3.2. Overall Fusion Rate Progression

**Figure 6:**
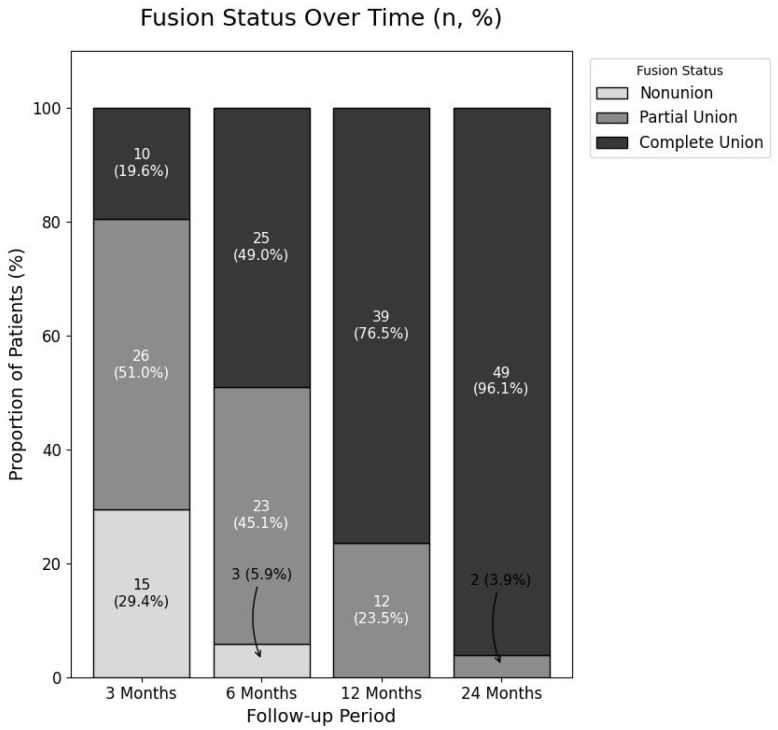
Progression of Radiological Fusion Status Over Time.

The overall radiological fusion status showed a clear progression towards solid union over the 24-month follow-up period (Figure 6). The rate of Complete Fusion (Grade 2) steadily increased from 19.6% at 3 months, to 49.0% at 6 months, 76.5% at 12 months, and ultimately reached 96.1% (49 of 51 patients; 95% CI, 86.5–99.5%) at the final follow-up. Notably, no cases of Non-union (Grade 0) were observed at or after the 12-month follow-up, leaving only two patients (3.9%) in a state of Partial Fusion at the study’s conclusion.

This 100% stacked bar chart illustrates the changes in fusion status at the 3, 6, 12, and 24-month follow-up points. The proportions of patients classified as non-union (Grade 0), partial fusion (Grade 1), and complete fusion (Grade 2) are represented by different shades. The numbers within each segment indicate the absolute number of patients (*n*) and the corresponding percentage (%). A clear, progressive increase in the rate of complete fusion is demonstrated, reaching 96.1% at the final follow-up.

### 3.3. Analysis of Fusion Location at 24 Months

The complete fusion rates at the 24-month follow-up were analyzed separately for the sagittal and coronal planes. In the sagittal plane, the complete fusion rate was highest in the posterior location (76.5%), followed by the intracage (62.7%) and anterior (31.4%) locations. A Cochran’s Q test confirmed a statistically significant difference among these three sites (*p* < 0.0001). Post hoc analysis revealed that fusion rates in the posterior and intracage locations were significantly higher than in the anterior location. In the coronal plane, the intracage location had the highest fusion rate (72.5%), compared to the left (29.4%) and right (21.6%) locations. This difference was also statistically significant (*p* < 0.0001, Cochran’s Q test), with post hoc tests showing that the intracage fusion rate was significantly higher than both the left and right sides. These analyses statistically demonstrate that successful bony fusion occurred predominantly in the posterior region on sagittal view and within the intracage region on coronal view.

### 3.4. Factors Influencing Fusion Outcomes

An analysis was performed to identify factors associated with the final fusion status at 24 months (Table 2). No statistically significant association was found between the final fusion outcome (complete vs. partial Fusion) and patient-related factors, including mean age (69.8 vs. 75.0 years, *p* = 0.679) and BMD T-score (−1.7 vs. −2.1, *p* = 0.561). Similarly, various surgery-related factors—such as cage height, lordotic angle, insertion site, and final cage position—did not show a significant influence on the 24-month fusion rate (all *p* > 0.05).

In contrast, the composition of the bone graft material was the only factor found to have a significant influence on the fusion outcome (Table 3). The ‘DBM with I-factor’ group demonstrated a significantly higher rate of achieving complete Fusion compared to the ‘DBM only’ group at all postoperative follow-up points: 3 months (*p* < 0.001), 6 months (*p* = 0.002), 12 months (*p* = 0.022), and at the final 24-month follow-up (*p* = 0.045). At the 24-month assessment, all 39 patients (100%) in the ‘DBM with I-factor’ group achieved complete Fusion, whereas 10 of the 12 patients (83.3%) in the ‘DBM only’ group achieved the same, with two patients remaining in a state of Partial Fusion.

**Figure 7:**
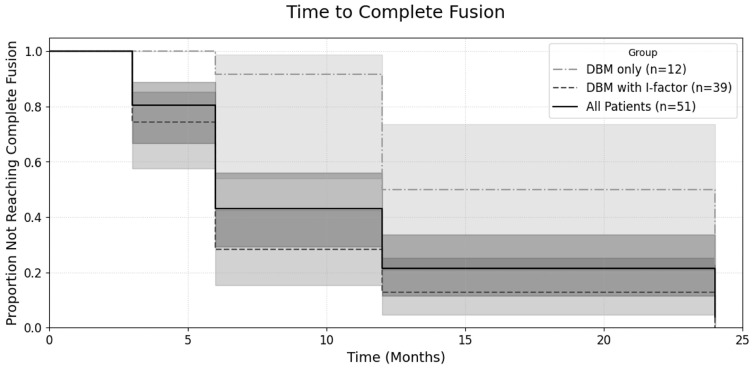
Kaplan–Meier curves for the time to complete fusion.

While comparisons at each individual follow-up point revealed higher fusion rates for the ‘DBM with I-factor’ group, to more comprehensively evaluate the overall progression of fusion over the entire follow-up period, a Kaplan–Meier analysis was performed (Figure 7). This analysis confirmed that the ‘DBM with I-factor’ group (*n* = 39) achieved complete Fusion at a significantly faster overall rate than the ‘DBM only’ group (*n* = 12). The difference between the survival curves was statistically significant (log-rank test, *p* < 0.001).

The plot shows the proportion of patients who had not yet achieved complete fusion over a 24-month follow-up period. The central lines represent the Kaplan–Meier estimates for each group: the solid line for all patients (*n* = 51), the dashed line for the DBM + I-factor group (*n* = 39), and the dash-dot line for the DBM only group (*n* = 12). Each corresponding shaded area represents the 95% confidence interval for that estimate. A log-rank test was used to compare the curves between the ‘DBM only’ and ‘DBM with I-factor’ groups, which showed a statistically significant difference (*p* < 0.001).

## 4. Discussions

The primary finding of this study is the high rate of radiological fusion following BESS-TLIF using the EIT Cellular Titanium cage. At the final 24-month follow-up, a complete fusion (Grade 2) rate of 96.1% was achieved. Our analysis also revealed that bony fusion occurred predominantly in the posterior and intracage regions, and that the addition of I-factor significantly accelerated the time to achieve solid fusion.

The 96.1% fusion rate in this study is consistent with the 95.2% pooled fusion rate for the BESS-TLIF technique reported in a recent meta-analysis [13]. Furthermore, this high fusion rate is comparable to the findings of a recent meta-analysis on conventional techniques, which reported fusion rates of 94.8% for MIS-TLIF and 93.9% for open TLIF [14]. This suggests that the BESS-TLIF technique, when combined with a porous titanium cage, can achieve a fusion success rate at least equivalent to that of more traditional and invasive approaches.

The high fusion rate in this study may also be attributable to the characteristics of the interbody cage. The banana-shaped design allows for a large footprint, maximizing contact with the biomechanically robust apophyseal ring of the vertebral endplate [15]. Moreover, the favourable outcomes in this study are likely attributable in large part to the characteristics of the interbody implant. The EIT cage is constructed from highly porous titanium, with a trabecular structure designed to mimic cancellous bone and facilitate osseointegration [16]. The clinical superiority of such materials over traditional PEEK is increasingly supported by high-level evidence; a recent meta-analysis confirmed that porous titanium cages are associated with significantly higher fusion rates and lower subsidence rates [17].

During our procedure, bone graft material was first placed into the prepared disc space, followed by the insertion of the interbody cage. This procedural sequence consequently leaves a relatively smaller volume of graft material in the posterior aspect of the segment. Despite this, a notable finding was that the rate of Complete Fusion in the posterior region (76.5%) was significantly higher than that in the anterior region. This finding is consistent with previous CT-based analyses of interbody fusion patterns, which have demonstrated that bone union tends to initiate at the posterior margin of the disc space [18]. Following stabilization with an interbody cage and pedicle screws, the posterior aspect of the disc space is subjected to significant compressive forces [19]. This concentrated mechanical stress is known to stimulate a robust bone formation response, which likely explains the observed fusion pattern [20].

Furthermore, the analysis of the coronal plane revealed another important fusion pattern: the rate of intracage fusion (72.5%) was significantly higher than the fusion rates in the lateral aspects of the disc space (29.4% on the left and 21.6% on the right). This finding is consistent with the biomechanical principles of interbody fusion. The interbody cage is designed to be the primary load-bearing structure, transmitting axial compressive forces through the centre of the vertebral endplates [21]. This creates an ideal mechanical environment for osteogenesis within and immediately around the cage, which also contains the most densely packed bone graft on an osteoconductive scaffold [22]. The lateral gutters, in contrast, are subjected to less direct compressive loading, which may explain the lower fusion rates observed in these areas [23].

The only factor found to have a statistically significant influence on fusion outcomes in this study was the composition of the bone graft material. The Kaplan–Meier analysis confirmed a significantly faster time to achieve solid fusion in the ‘DBM with I-factor’ group compared to the ‘DBM only’ group (log-rank test, *p* < 0.001). Furthermore, at the final 24-month follow-up, the Complete Fusion rate was 100% in the I-factor group versus 83.3% in the ‘DBM only’ group. This finding can be attributed to the different biological mechanisms of the graft materials. DBM primarily acts as an osteoconductive scaffold, providing a passive framework for bone growth. In contrast, I-factor is a synthetic peptide (P-15) that functions as an osteoinductive agent. Specifically, the P-15 peptide mimics the binding site of Type I collagen, which actively attracts osteoprogenitor cells and stimulates their attachment and proliferation to enhance the cascade of new bone formation [8]. While other potent osteoinductive agents, such as recombinant human bone morphogenetic protein-2 (rhBMP-2), are available, their use in MISS procedures has been linked to significant complications like ectopic bone formation and vertebral osteolysis, as noted in our methods [5,8]. Our results, which align with the known biological activity of P-15, suggest that the selective addition of this synthetic peptide, which offers a favourable safety profile, can be a valuable strategy to accelerate and ensure a higher probability of successful arthrodesis in BESS-TLIF procedures.

The present study has several distinct features. First, the study cohort was highly homogeneous. By including only patients who underwent single-level fusion with a single, specific type of interbody cage, we minimized the confounding effects of surgical and implant-related variables. This allows for a more direct assessment of the radiological outcomes of the BESS-TLIF procedure when performed under these standardized conditions. Second, our study utilized CT scans for fusion assessment up to a 24-month follow-up. The use of CT, which is considered the gold standard for evaluating bony fusion, at a long-term time point provides a more accurate and reliable assessment of fusion status compared to studies with shorter follow-up periods or those relying solely on plain radiographs.

This study has several limitations that should be acknowledged. First, its retrospective design is subject to inherent selection bias and potential inconsistencies in the collected data. Second, the relatively small sample size and the single-centre nature of the study may limit the generalizability of our findings to a broader patient population. Third, this was a single-arm study without a direct comparative control group, such as patients undergoing open TLIF or implantation with a different type of cage. Finally, our analysis focused exclusively on radiological outcomes, and a correlation with clinical results, such as pain and functional scores, was not performed. Therefore, the high radiological fusion rate reported here does not necessarily translate to a superior clinical outcome. Furthermore, it is important to note that four patients (7.3% of the initial cohort) were excluded from the final fusion analysis due to significant cage subsidence [6]. While these cases were excluded because severe subsidence confounds the accurate radiological assessment of fusion, subsidence itself can be considered a form of mechanical or biological failure. Therefore, the reported fusion rate of 96.1% should be interpreted with caution, as it represents a per-protocol outcome and may potentially overestimate the overall success rate of the procedure.

## 5. Conclusions

In conclusion, this study demonstrates that single-level BESS-TLIF using a banana-shaped, porous titanium cage can achieve a very high radiological fusion rate of 96.1% (95% CI, 86.5–99.5%) at the 24-month long-term follow-up. Successful bony fusion occurred predominantly in the posterior and intracage regions of the vertebral segment, suggesting the importance of a stable biomechanical environment. Furthermore, the addition of an osteoinductive biologic agent (I-factor) was found to significantly accelerate the time to achieve fusion. Therefore, the results of this study, with its strictly controlled variables, support that BESS-TLIF, when combined with a porous titanium implant of this specific design, is an effective minimally invasive treatment method capable of achieving reliable bony fusion.

## Figures and Tables

**Table 1:** Baseline Demographic and Surgical Characteristics (*n* = 51).

	All Patients (*n* = 51)
Age (years), mean (SD)	70.0 (9.7)
BMD, mean (SD)	−1.7 (1.1)
Sex, *n* (%)	
Male	22 (43.1%)
Female	29 (56.9%)
Fusion Material, *n* (%)	
DBM	12 (23.5%)
DBM with I-factor	39 (76.5%)
Surgical Level, *n*	
L1–2	1 (2.0%)
L2–3	2 (3.9%)
L3–4	8(15.7%)
L4–5	27 (52.9%)
L5–S1	13 (25.5%)
Cage Height (mm), *n* (%)	
9	1 (2.0%)
10	11 (21.6%)
11	11 (21.6%)
12	20 (39.2%)
13	8 (15.7%)
Cage Angle (°), *n* (%)	
8	38 (74.5%)
12	13 (25.5%)

Values are presented as mean (standard deviation) or *n* (%). SD: Standard Deviation; BMD: Bone Mineral Density; DBM: Demineralized Bone Matrix.

**Table 2:** Analysis of Factors Associated with Fusion Status at 24 Months.

	Complete Fusion (*n* = 49)	Partial Fusion (*n* = 2)	*p*-Value
Age (years)	69.8 (9.7)	75.0 (11.3)	0.679
BMD (T-score)	−1.7 (1.1)	−2.1 (0.6)	0.561
Cage Height (mm)	11.5 (1.1)	10.5 (0.7)	0.171
Cage Angle (°)	9.0 (1.7)	10.0 (2.8)	0.422
Insertion Site			0.353
Right	29 (59.2)	0 (0.0)	
Left	20 (40.8)	2 (100.0)	
Cage Position			0.981
Centre	23 (46.9)	1 (50.0)	
Anterior	22 (44.9)	1 (50.0)	
Anterior–Posterior	2 (4.1)	0 (0.0)	
Posterior	2 (4.1)	0 (0.0)	
Fusion Material			
DBM only	10 (20.4)	2 (100.0)	
DBM with I-factor	39 (79.6)	0 (0.0)	0.045

Values are presented as mean (standard deviation) or *n* (%). Continuous variables were compared using the Kruskal–Wallis H test, and categorical variables were compared using Fisher’s exact test. *p* < 0.05 was considered statistically significant. BMD: Bone Mineral Density; DBM: Demineralized Bone Matrix.

**Table 3:** Comparison of Fusion Progression by Bone Graft Material.

Follow-Up	Fusion Status	DBM Only (*n* = 12)	DBM with I-Factor (*n* = 39)	*p*-Value	
3 Months	Nonunion	11	4	<0.001	
	Partial Union	1	25		
	Complete Union	0	10		
6 Months	Nonunion	2	1	0.002	
	Partial Union	9	14		
	Complete Union	1	24		
12 Months	Nonunion	0	0	0.022	
	Partial Union	6	6		
	Complete Union	6	33		
24 Months	Nonunion	0	0		0.045
	Partial Union	2	0		
	Complete Union	10	39		

Values are presented as *n* (%). *p*-values were obtained using Fisher’s exact test to compare the distribution of fusion grades between the two groups at each time point. *p* < 0.05 was considered statistically significant. DBM: Demineralized Bone Matrix.

## Data Availability

The data presented in this study are available on request from the corresponding author. The data are not publicly available due to patient privacy and ethical restrictions.
